# Managing Complications of Patellar Fracture Hardware Removal

**DOI:** 10.7759/cureus.12364

**Published:** 2020-12-29

**Authors:** Blake Callahan, Patricia Baumann

**Affiliations:** 1 Orthopaedic Surgery, University of Central Florida (UCF) College of Medicine, Orlando, USA; 2 Orthopaedic Surgery, C.W. Bill Young Department of Veterans Affairs Medical Center, Saint Petersburg, USA

**Keywords:** orthopedic infections, retained hardware, patellar fracture, orif, orthopedics and trauma, basic orthopedic sciences

## Abstract

A simple surgical procedure is not without the risk of complications and when removing hardware from the bone, such as a previous patella fracture hardware, the surgeon must be well aware of this potential. Here we present the case of a 71-year-old male who presented for removal of retained hardware of united right patella fracture with overlying skin complications. Surgical intervention was uneventful. On post-operative day 1, the patient suffered a fall while using the restroom unassisted with a subsequent large amount of bleeding from the incision site. X-rays demonstrated a new displaced inferior pole patellar fracture. The patient returned to the operating room for debridement and repair of this new patellar fracture with primary closure. Intraoperative cultures of initial operative site were positive for Corynebacterium and Staphylococcus epidermidis. The patient was then started on intravenous Vancomycin based on culture sensitivities. The patella fixation/repair was protected with a knee immobilizer as there was increased risk of falls due to his age/underlying medical condition. This case discusses recommendations and guidelines for preventing and managing these various postoperative complications.

## Introduction

Patellar fractures are common with an incidence reported in 1% of all skeletal injuries with a male to female ration of 2:1 with the most prevalent age range being between 20-50 years [1]. Direct trauma through motor vehicle accidents or falls are common etiologies. Patellar fractures are classified by location, displacement, and orientation, and are characterized as nondisplaced, displaced, transverse, pole or sleeve (upper or lower), vertical, marginal, osteochondral, or comminuted [1]. The management of these fractures varies depending on fracture classification and other co-morbidities. Treatment ranges from non-operative management, open reduction with internal fixation (ORIF), to partial or total patellectomy [1]. When operative management is undertaken, the most common procedure is ORIF, from which complications can arise. These complications are common and primarily include failure of hardware, nonunion, new fracture, infection, and continued pain. It has been indicated that around 22% of ORIF of patella fractures present with hardware failure, within the first six weeks postoperatively. Furthermore up to 50% of patients will require removal of hardware in the future [2]. Preventing and managing complications of initial ORIF and subsequent procedures should be given careful consideration as highlighted in this case report.

## Case presentation

A 71-year-old male presented with 15-year history of anterior right knee pain following ORIF of patella fracture. The patient had a comminuted patellar fracture treated with horizontally placed cannulated screws with cable fixation traversing the screws. He explained that at some point shortly after surgery, the cable traversing the cannulated screws broke and had gradually been working its way to protruding through the skin of the medial aspect of the knee. He had also developed 5/10 medial based pain. He was examined and diagnosed with retained hardware with possible infection.

The patient presented with a complex medical history including hyperlipidemia, vitamin D deficiency, vitamin B12 deficiency, chronic bilateral sciatica, tobacco dependence, obesity, coronary artery disease with myocardial infarction, peripheral artery disease, hypertension and diabetes mellitus. His allergies included gatifloxacin and atorvastatin. His family history was unrevealing. His surgical history included the original ORIF of the patella and one subsequent procedure including open debridement of a patellar cyst, one year prior to current presentation.

This patient was a current smoker who smoked half a pack a day with a history of fifty pack years. He denied alcohol or drug consumption. He is divorced, retired, and lives alone. Due to history of diabetes he maintained a diabetic diet.

Initial vitals on presentation included: Temperature 98.5F, heart rate 75, respiratory rate 18, blood pressure 150/70 mmHg, weight 214.6 lbs, body mass index 32.4.

Preoperatively the patient’s physical exam of the right knee was as follows: No muscle atrophy appreciated; 1 cm dark wheal located over the superomedial aspect of the right knee. Range of motion was from 5 degrees to 130 degrees with moderate pain. No crepitus to palpation. No calf tenderness, negative Homans sign. Sensation intact with no numbness or paresthesia, femoral, popliteal, dorsalis pedis, and posterior tibial pulses 2+. Strength testing demonstrated flexion and extension rated 4/5, plantarflexion and dorsiflexion of the ankle rated 5/5.

**Figure 1:**
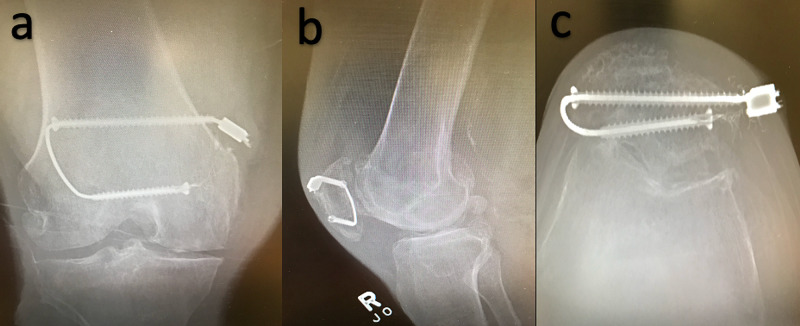
X-ray views on presentation Anteroposterior (AP) (a), lateral (b), and sunrise (c) X-ray views of the right knee demonstrated two horizontally oriented cannulated screws traversed by metal cable with segment of cable abutting the skin over the superomedial knee. The superior screw appears with step off medial bone surface.

Diagnostic data were as follows: Laboratory data including complete blood count (CBC), basic metabolic panel (BMP), prothrombin time (PT), partial thromboplastin time (PTT), international normalized ratio (INR), and urinalysis were within normal limits with the exception of hemoglobin A1c which was elevated at 7.1. Preoperative electrocardiogram (EKG) demonstrated multiple premature ventricular contractions (PVCs) and cardiac clearance was obtained. X-rays of the right knee are shown in Figure 1.

Removal of retained hardware

The patient underwent removal of two cannulated screws with a broken traversing cable without complication. A skin wheal where the cable was found to be completely penetrating through the skin was removed. Culture and sensitivity of skin wheal and bone tunnels were obtained intra-operatively. Skin was closed and the patient was sent to the postoperative recovery room in stable condition.

Continued clinical course

**Figure 2:**
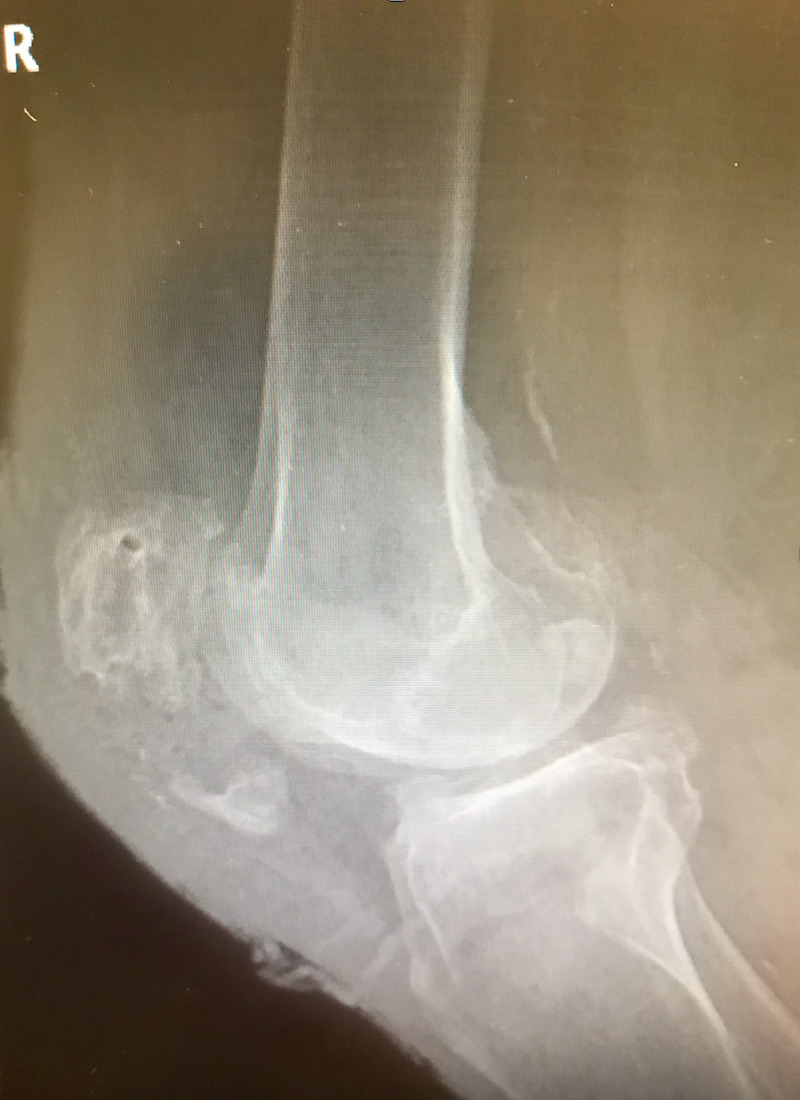
Lateral X-ray after fall Lateral X-ray view of the right knee following fall with wound dehiscence demonstrates new displaced horizontal patellar fracture of the inferior pole of the patella. Patella alta is noted.

On post-operative day 1, the patient reported one episode of a fall after trying to ambulate without assistance where he described a sensation of weakness. The patient denied head injury or loss of consciousness. Following this unwitnessed fall, a head CT was ordered but was refused by the patient. Later, on post-operative day 1, the patient experienced a second witnessed fall with hyperflexion of knee and wound dehiscence. Telemetry at time of fall detected sinus bradycardia. Repeat X-rays of the right knee suggested a new horizontal inferior patellar fracture through the previous inferior cannulated screw site as shown in Figure 2.

On exam, the patient had loss of ability to perform straight leg raise indicating failure of the extensor mechanism. The incision was actively bleeding, and the deep fascial sutures had been compromised. In addition to this new injury, gram stain from the removal of the original hardware grew Corynebacterium and Staphylococcal species.

**Figure 3:**
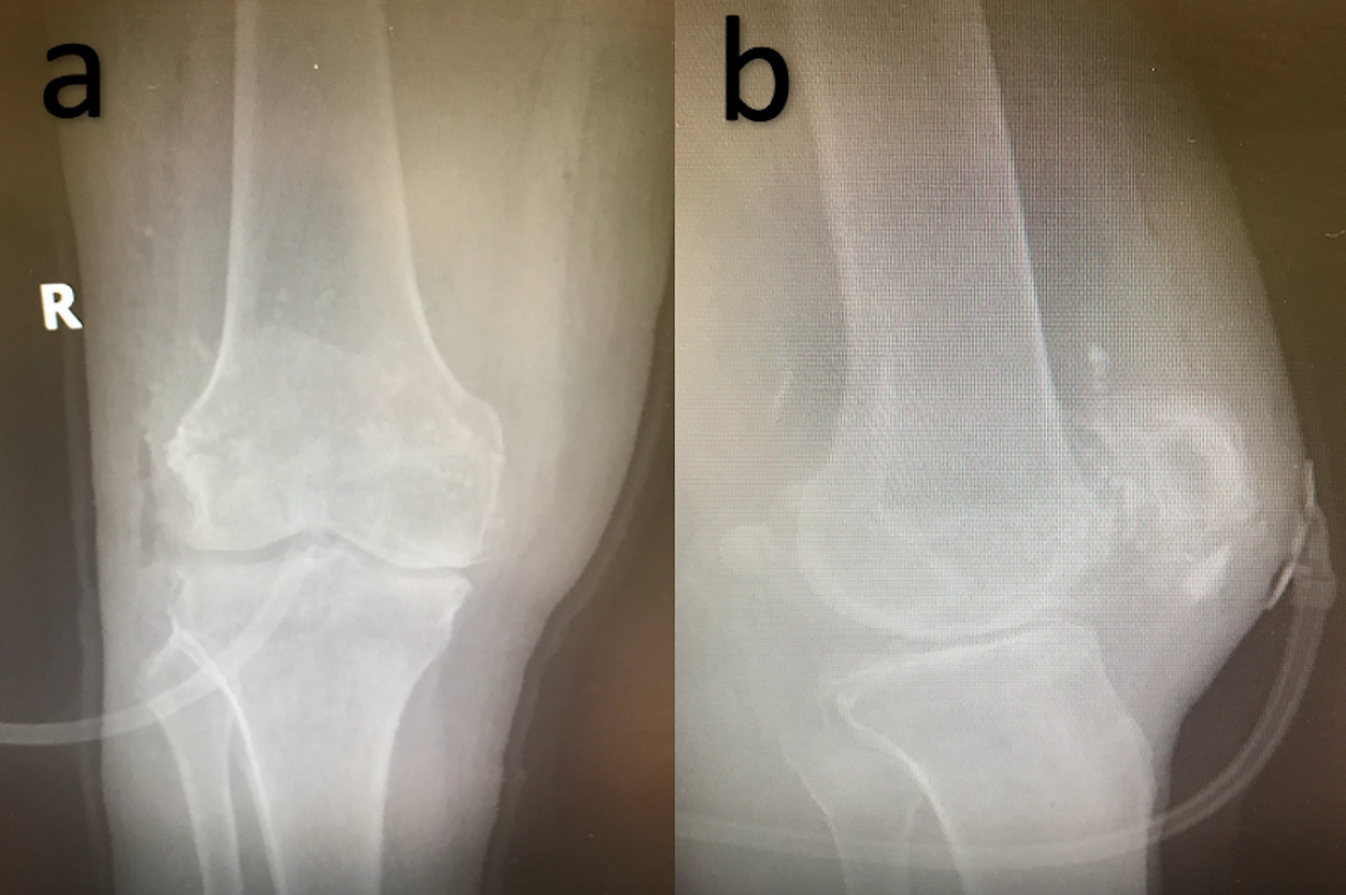
Post fracture repair X-rays Anteroposterior (AP) (a) and lateral (b) X-rays demonstrate successful repair of patellar fracture and retinaculum. Negative pressure dressing is observed in place.

The patient was once again taken to the operating room for repair of the patellar fracture and retinaculum. Bio-absorbable antibiotic beads were concurrently placed for management of infection. The site was closed by primary closure and a negative pressure wound dressing was applied. Figure 3 shows postoperative X-rays. IV vancomycin was initiated once cultures confirmed the identity of the gram-positive rods as Corynebacterium. A knee immobilizer was applied to protect the surgical repair in the face of potential recurrent falls.

## Discussion

The removal of retained hardware for patellar fractures is relatively commonplace, but there are risks of undertaking such an endeavor. To reduce the risk of complications, one begins with proper initial treatment of the original fracture. Patellar fractures can be managed with nonoperative treatment if the fracture is deemed stable without displacement greater than 2 mm [1]. Nonoperative management can include knee immobilizers, braces, casting, or splinting for 6-8 weeks in addition to weightbearing as tolerated. According to the literature the most common method of fixation is to run at least two K wires or cables perpendicular to the fracture site [1]. If this were the case the two horizontally oriented cannulated screws in the original plain films suggest a vertical fracture or a comminuted fracture. The original operative report establishes the fracture as being severely comminuted but had the original fracture been vertical, then the fracture may have been better suited for nonsurgical management, thus preventing the complication of skin penetrating retained hardware as seen in this patient. Other possible complications of ORIF of a patella fracture include anterior knee pain, weakness, osteonecrosis, nonunion of fragments, stiffness, and infection [1].

When operative management is required, intraoperative considerations must be evaluated. As discussed above, the primary method of fixation of a patellar fracture, is the use of at least two parallel wires or cannulated screws running perpendicular to the fracture line and placed under tension. Multiple Cerclage wires and plate fixation are also possible methods to achieve fixation [3]. In the cases of small fragment fracture, partial patellectomy is possible but has less favorable outcomes than fixation. In cases of severe comminution, where fixation is not possible, complete patellectomy is required with outcomes being least favorable [1].

When cannulated screws are used, it is necessary to avoid overhanging screws over the edge of the bone and to place the construct under appropriate tension being careful not over tighten [3]. Failure to achieve these parameters may lead to failure of the hardware, as seen in our case.

In this case of the skin penetrating retained hardware, the consideration of infection must also be evaluated. Cultures of the hardware should be obtained and followed closely as done in this case. If there is moderate to strong suspicion of infection, empiric antibiotic therapy should be initiated. When cultures return, appropriate IV antibiotic treatment should be continued for 3-6 weeks [4]. Furthermore, it may be preferred to use monofilament sutures and avoid braided cabling systems, which can act as a nidus for infection to persist [5].

The postoperative period, for any procedure, can be a difficult time to manage and prevent complications. For hardware removal such as in our patient's initial procedure, the complication of a fall was an inherent possibility. Patients should be screened pre-operatively and postoperatively for fall risk assessment [6]. There are several screening assessments that can reliably predict fall risk and take into consideration variables such as age, mental status, neurological function, and motor strength. It is suggested that fall risk assessments should be performed at short intervals in the acute care setting regardless of medical indication for admission [6]. When fall risk is identified as being moderate or greater, precautions should be taken to minimize the risk of patient injury.

Typically, after ORIF of the patella, the knee can be placed in a knee immobilizer or range of motion brace locked or restricted in extension to prevent fall due to weakness [1]. In our case, a knee immobilizer was applied to protect the fracture repair due to patient noncompliance and risk for future falls. Range of motion should also be restricted for the first six weeks following the procedure [1].

## Conclusions

This case highlights the complications of hardware failure, recurrent pain, new fracture, and infection following ORIF of the patella which illustrates just some of the complications that can arise from this procedure. Careful considerations should be given preoperatively, intraoperatively and postoperatively using established literature and guidelines. If evaluated properly, such considerations can help prevent and manage complications and can lead to good clinical outcomes.
